# Angiopoietin-like 4 Mediates Colonic Inflammation by Regulating Chemokine Transcript Stability via Tristetraprolin

**DOI:** 10.1038/srep44351

**Published:** 2017-03-13

**Authors:** Terri Phua, Ming Keat Sng, Eddie Han Pin Tan, Dickson Shao Liang Chee, Yinliang Li, Jonathan Wei Kiat Wee, Ziqiang Teo, Jeremy Soon Kiat Chan, Maegan Miang Kee Lim, Chek Kun Tan, Pengcheng Zhu, Velmurugesan Arulampalam, Nguan Soon Tan

**Affiliations:** 1 School of Biological Sciences, Nanyang Technological University, 60 Nanyang Drive, Singapore 637551, Singapore; 2 Department of Microbiology, Tumor and Cell Biology, Karolinska Institutet, Nobels väg 16, Stockholm 17177, Sweden; 3 Lee Kong Chian School of Medicine, Nanyang Technological University, 50 Nanyang Drive, Singapore 639798, Singapore; 4 Institute of Molecular Cell Biology, 61 Biopolis Drive, Proteos, Agency for Science Technology & Research, Singapore 138673, Singapore; 5 KK Research Centre, KK Women’s and Children Hospital, 100 Bukit Timah Road, Singapore 229899, Singapore

## Abstract

Many gastrointestinal diseases exhibit a protracted and aggravated inflammatory response that can lead to hypercytokinaemia, culminating in extensive tissue damage. Recently, angiopoietin-like 4 (ANGPTL4) has been implicated in many inflammation-associated diseases. However, how ANGPTL4 regulates colonic inflammation remains unclear. Herein, we show that ANGPTL4 deficiency in mice (ANGPTL4^−/−^) exacerbated colonic inflammation induced by dextran sulfate sodium (DSS) or stearic acid. Microbiota was similar between the two genotypes prior DSS challenge. A microarray gene expression profile of the colon from DSS-treated ANGPTL4^−/−^ mice was enriched for genes involved in leukocyte migration and infiltration, and showed a close association to inflamed ulcerative colitis (UC), whereas the profile from ANGPTL4^+/+^ littermates resembled that of non-inflamed UC biopsies. Bone marrow transplantation demonstrates the intrinsic role of colonic ANGPTL4 in regulating leukocyte infiltration during DSS-induced inflammation. Using immortalized human colon epithelial cells, we revealed that the ANGPTL4-mediated upregulation of tristetraprolin expression operates through CREB and NF-κB transcription factors, which in turn, regulates the stability of chemokines. Together, our findings suggest that ANGPTL4 protects against acute colonic inflammation and that its absence exacerbates the severity of inflammation. Our findings emphasize the importance of ANGPTL4 as a novel target for therapy in regulating and attenuating inflammation.

An aggravated inflammatory response is a common feature of many gastrointestinal disorders, such as inflammatory bowel diseases, enteritis, and colitis. Many of these conditions are caused by changes in dietary fat intake, the ingestion of bacteria-contaminated food and water, and certain chemicals. These insults trigger an inflammatory response by inducing the recruitment of macrophages to the site of inflammation to combat pathogens, neutralize harmful immunogens and promote tissue repair^1^. However, a protracted inflammatory response can cause tissue damage and lead to hypercytokinaemia, which is a potentially fatal immune reaction. Immune cell infiltration into the site of damage is highly regulated by chemotactic factors, such as macrophage inflammatory protein 1α and chemokine (C-C motif) ligand 2 (CCL2)^2^,^3^. As the initial cellular barrier that encounters lumenal insults, intestinal and colonic epithelia play important roles in the early recruitment of inflammatory cells to the mucosa. Epithelial cells are a major source of chemoattractants, and epithelial chemokine production has been proposed as a key target of future therapies for gastrointestinal disorders^4^. However, much remains to be understood about the mechanisms that regulate the levels of these chemokines in the gastrointestinal and colonic tracts.

Angiopoietin-like 4 (ANGPTL4) is a matricellular protein that has been implicated in many inflammation-associated diseases^5^. Native full-length ANGPTL4 (fANGPTL4) is proteolytically cleaved into two functionally distinct isoforms: the N-terminal domain (nANGPTL4) inhibits lipoprotein lipase (LPL) and directly regulates energy homeostasis, while the C-terminal domain (cANGPTL4) has been implicated in various processes such as cancer metastasis, skin wound and pulmonary inflammation^6^,^7^,^8^. Diabetic wounds show low endogenous cANGPTL4 levels and have been associated with an elevated F4/80^+^ macrophage population at the wound site. The infiltration of F4/80^+^ macrophages was reduced upon treatment of diabetic wounds with recombinant cANGPTL4 when compared with saline^9^. ANGPTL4 can also protect against the severe pro-inflammatory effects of saturated fat by inhibiting fatty acid uptake by mesenteric lymph node macrophages^10^. Similarly, ANGPTL4 confers protective effects against the development of atherosclerosis^11^, which has been associated with atherogenesis and macrophage polarization^12^. ANGPTL4 has also been identified as an angiogenic mediator in arthritis^13^. ANGPTL4 has been observed to exacerbate influenza-associated inflammation through IL-6–Stat3 signaling in the lung^14^. Furthermore, serum ANGPTL4 was associated with the C-reactive protein level in type II diabetic patients, suggesting that ANGPTL4 may be involved in the progression of inflammation during metabolic syndrome^15^. Thus, ANGPTL4 may exert both anti- and pro-inflammatory effects in a context-dependent manner. Despite numerous reports of the role of ANGPTL4 in inflammation, the mechanisms whereby ANGPTL4 modulates inflammation in various diseases remain largely unclear.

Herein, we describe an anti-inflammatory role for colonic ANGPTL4 in dextran sulfate sodium salt (DSS)-induced colitis and dietary stearic acid (SA) intake *in vitro* and *in vivo.* We showed that the microbiota was similar between ANGPTL4^+/+^ and ANGPTL4^−/−^ mice at steady states, but with perturbation such as DSS treatment some differences in microbiota community become accentuated. Bone marrow transplantation and microarray analysis confirmed the intrinsic role of colonic ANGPTL4 in regulating leukocyte infiltration during DSS-induced inflammation, and thus the colonic inflammatory landscape. The underlying mechanism involves the regulation of tristetraprolin (TTP or ZFP36), an mRNA-binding protein that is involved in chemokine destabilization, by ANGPTL4 via activation of CREB and NF-κB transcription factors.

## Results

### ANGPTL4 reduces DSS- and saturated fat-induced colonic inflammation

**Figure 1:**
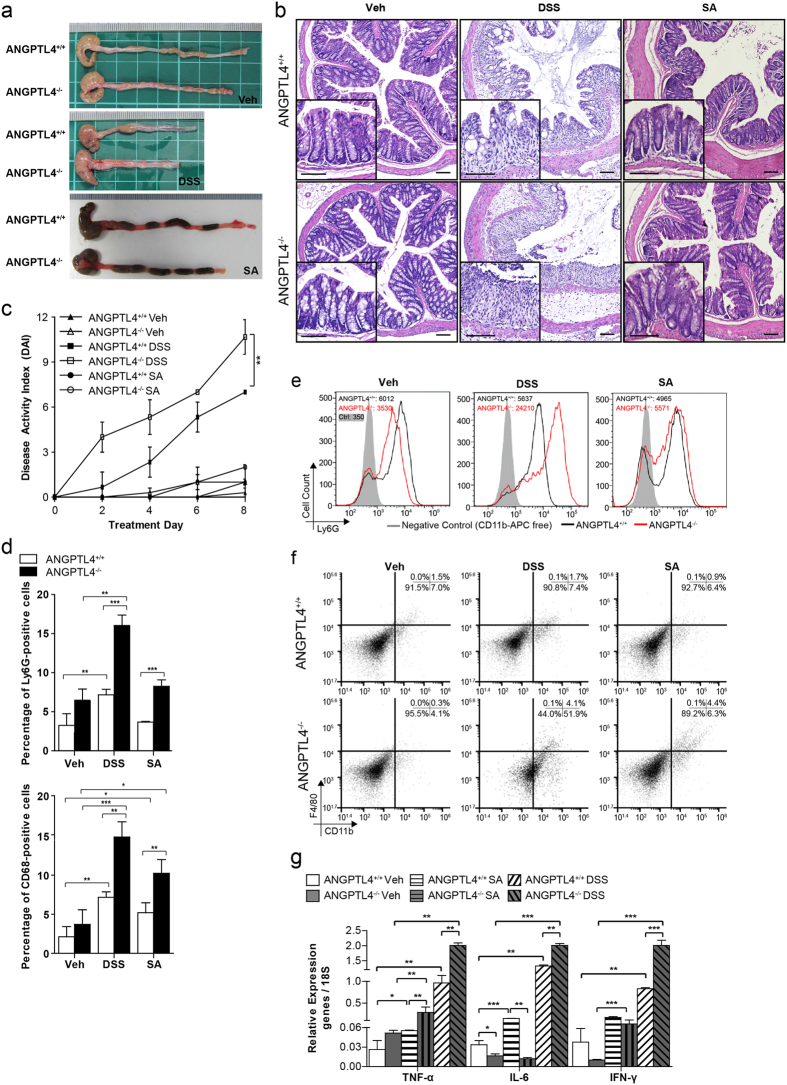
ANGPTL4 attenuates colonic inflammation. (**a–e)** Images of colon samples (**a)** Representative colon sections stained with hematoxylin and eosin (H&E) (**b**). (**c**) Disease activity index (DAI) of mice assessed every alternate day over the treatment regimes. Scoring criteria can be found in Supplementary Table S1. (**d**) Percentage of Ly6G^+^ and CD68^+^ cells per field of view. Total DAPI-stained nuclei were taken as the total cell number. Microscopic views from 5 different sections were numerated. (**e)** Representative FACS histograms for Ly6G expression (neutrophils) from ANGPTL4^+/+^ and ANGPTL4^−/−^ littermates for the indicated treatments. (**f**) Representative FACS analysis of the colons from ANGPTL4^+/+^ and ANGPTL4^−/−^ mice double stained for F4/80 (macrophages) and CD11b (macrophages, monocytes, granulocytes, NK cells, dendritic cells) for each of the treatments. (**g**) Relative mRNA levels of pro-inflammatory cytokines (TNF-α, IL-6 and IFN-γ) from ANGPTL4^+/+^ and ANGPTL4^−/−^ mice at the endpoint. For the Veh, DSS and SA groups, n = 20 mice were used for each treatment. The Mann–Whitney U test was used. *p < 0.05; **p < 0.01.

We first characterized the intestinal and colonic tract of unchallenged ANGPTL4-knockout (ANGPTL4^−/−^) and wild-type (ANGPTL4^+/+^) mice. There was no significant difference in body weight, colon length, disease activity index (DAI), endpoint macroscopic scores or histological scores between the genotypes (Fig. 1a and Supplementary Fig. S1a,c). Detailed examination revealed that ANGPTL4^−/−^ mice exhibited an increased muscularis thickness and shorter colonic villus length than ANGPTL4^+/+^ littermates (Fig. 1b, Supplementary Fig. S1d,e). To gain insights into the role of ANGPTL4 in acute colonic inflammation, we challenged ANGPTL4^−/−^ and ANGPTL4^+/+^ mice to either 5% DSS or 15% (w/w) stearic acid (SA) for 8 days.

ANGPTL4^−/−^ mice receiving DSS lost significantly more weight compared with ANGPTL4^+/+^ mice from day 6 onwards (Supplementary Fig. S1a). DSS-treatment resulted in a significant increase in both the DAI and endpoint macroscopic score (Fig. 1c and Supplementary Fig. S1b) as well as a reduction in the ANGPTL4^+/+^ colonic villus length, whereas there was no significant reduction observed in ANGPTL4^−/−^ mice compared with their cognate controls (Fig. 1b and Supplementary Fig. S1e). Histological analysis of the DSS-challenged ANGPTL4^−/−^ colon sections revealed a severe loss of epithelial integrity and a massive infiltration of immune cells into the lamina propria when compared with cognate ANGPTL4^+/+^ colon (Fig. 1b). Indeed, we observed a higher number of Ly6G^+^ neutrophils and CD68^+^ macrophages in the colons of DSS-treated ANGPTL4^−/−^ mice (Fig. 1d and Supplementary Fig. S1f). FACS analysis also confirmed the higher number of Ly6G^+^, CD11b^+^ and F4/80^+^ cells in ANGPTL4^−/−^ mice compared to ANGPTL4^+/+^ mice (Fig. 1e,f). Such differences were not attributed to an increase in apoptotic cells between the genotypes, as indicated by TUNEL-positive cells and the expression of cleaved caspase 3 (Supplementary Fig. S1g). Consistent with the elevated inflammation status, we also detected higher levels of TNF-α, IL-6 and IFN-γ mRNA transcripts (Fig. 1g).

As expected, stearic acid induced a more subdued inflammatory response compared to the DSS challenge (Fig. 1g). No significant differences in weight gain, blood glucose levels, DAI, endpoint macroscopic scores or histological scores were observed between ANGPTL4^−/−^ and ANGPTL4^+/+^ mice on the SA diet compared with mice fed a normal chow diet (Fig. 1c and Supplementary Fig. S1a–c,h). ANGPTL4^+/+^ mice on an SA diet exhibited a longer colon, a thicker muscularis wall and a shorter colonic villus length at the endpoint compared with mice fed a standard diet (Fig. 1a,b and Supplementary Fig. S1c,d). Immunofluorescence staining showed a similar elevated number of Ly6G^+^ neutrophils and CD68^+^ macrophages in ANGPTL4^−/−^ colon (Fig. 1d and Supplementary Fig. S1f). FACS analysis also detected an elevated number of Ly6G^+^, CD11b^+^ and F4/80^+^ cells in ANGPTL4^−/−^ mice (Fig. 1e,f). Together, our data demonstrated that the absence of ANGPTL4 enriched for a subset of the immune cell population not limited to neutrophils, macrophages and eosinophils during inflammation. Altogether, our observations are consistent with a model whereby ANGPTL4 attenuates colonic inflammation in response to inflammatory stimuli.

### ANGPTL4 deficiency does not influence colonic commensal microbiota at steady state

**Figure 2:**
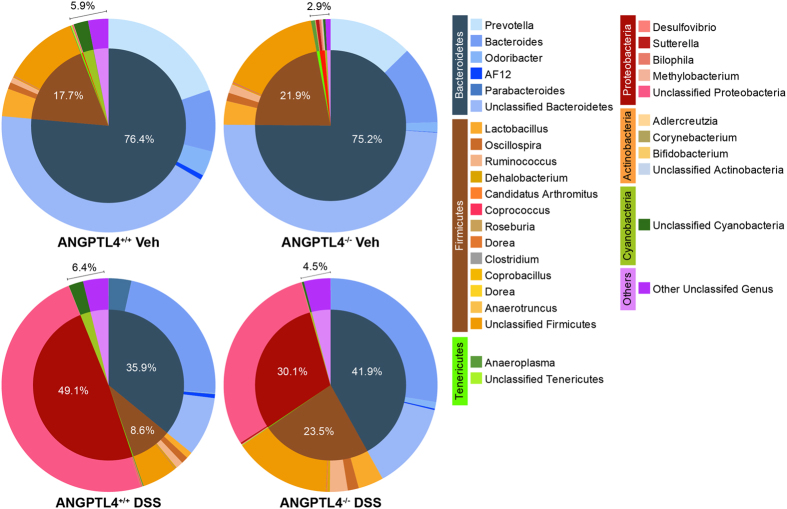
ANGPTL4 deficiency does not influence commensal microbe composition. Pie charts showing the commensal microbe landscape at steady state (*upper panel*) and after DSS treatment (*lower panel*) of age- and gender- matched ANGPTL4^+/+^ and ANGPTL4^−/−^ littermates. Values in the charts denote mean percentage of abundance. n = 6 mice per group.

Several reports have suggested that dysbiosis aggravates host inflammatory and metabolic diseases^16^,^17^,^18^,^19^. Studies also suggested that a subset of microbes could regulate ANGPTL4 expression in the gut in response to changes in energy demands^20^,^21^,^22^. To examine if the exacerbated colonic inflammatory response in ANGPTL4^−/−^ mice could be attributed to microbe composition, we performed 16S metagenomics sequencing using the V4 region to identify microbe abundance between ANGPTL4^+/+^ and ANGPTL4^−/−^ littermates before and after DSS-induced inflammation. Redundancy analysis showed that genotype and inflammation amounted to 41.2% of the total variation observed between microbial compositions in the colon. Genotype could only explain for 10.7% of the differences observed, while inflammation amounted to at least 31.1%. The steady state phylogenetic microbe composition was predominated by Firmicutes and Bacteriodetes, which did not differ much between both ANGPTL4^+/+^ and ANGPTL4^−/−^ mice before DSS treatment (Fig. 2). As expected, DSS-induced inflammation caused a sharp decrease in Bacteroidetes and a concomitant increase in Firmicutes and Proteobacteria. We found that Firmicutes were more abundant in inflamed ANGPTL4^+/+^ mice, while Proteobacteria was more prevalent in ANGPTL4^−/−^ mice (Fig. 2). Collectively, our data showed that microbe composition was similar between ANGPTL4^+/+^ and ANGPTL4^−/−^ mice at steady state, but with perturbation such as DSS treatment, some differences in the microbiota community become accentuated.

### Intrinsic role of colonic ANGPTL4 in leukocytes infiltration during DSS-induced inflammation.

**Figure 3:**
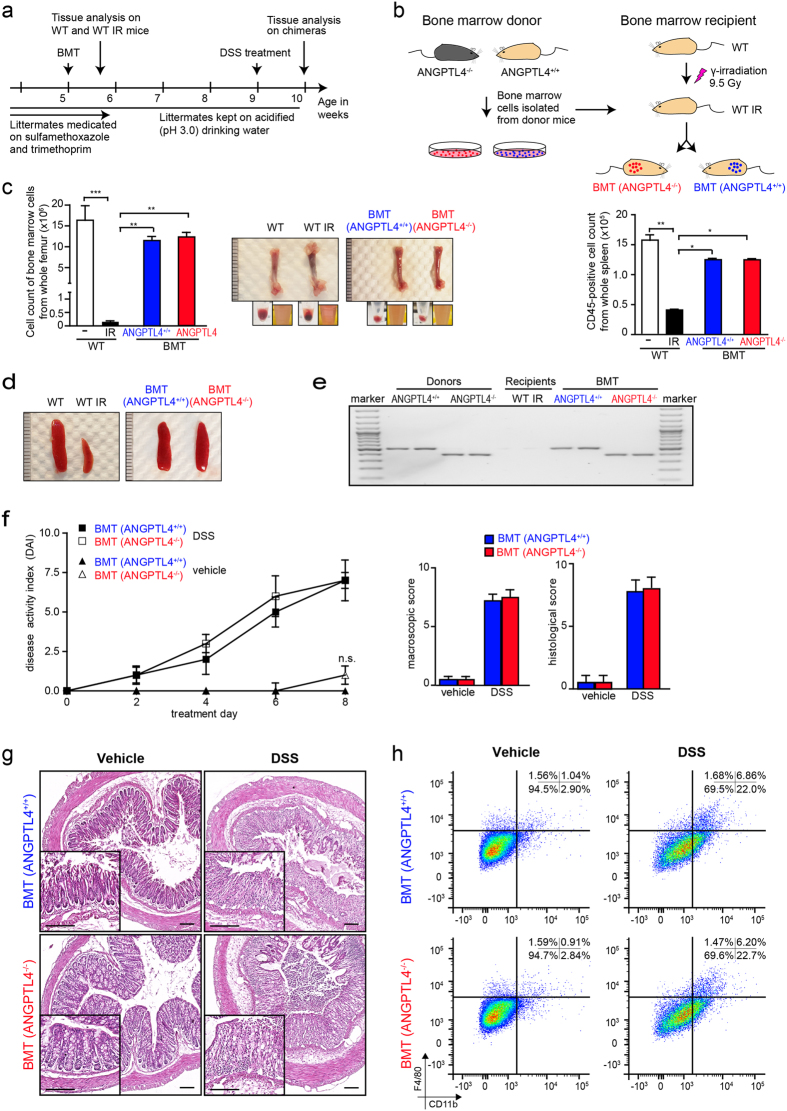
Intrinsic role of colonic ANGPTL4 in leukocytes infiltration during DSS. (**a**,**b)** Graphical illustrations depicting the experimental setup for bone marrow transplantation (BMT). CD4+ and CD8+ depleted bone marrow cells from age- and gender-matched ANGPTL4^+/+^ or ANGPTL4^−/−^ donors were transplanted into γ-irradiated ANGPTL4^+/+^ wild-type recipient (WT IR). Chimera BMT ANGPTL4^+/+^ and BMT ANGPTL4^−/−^ denote WT IR transplanted with bone marrow cells from ANGPTL4^+/+^ and ANGPTL4^−/−^ donor, respectively. Graph showed the number of CD45^+^ cells from spleen of indicated mice (Mean ± S.D.; n = 6 mice per group). (**c**,**d)** Representative images of the femur (**c**) and spleen (**d**) from indicated mice (WT and WT IR) and chimeras (BMT ANGPTL4^+/+^ and BMT ANGPTL4^−/−^). Inserts in (**c**) showed the bone marrow cell pellet before and after red blood cell lysis. Graph in (c; *left panel*) showed the number of bone marrow cells from femur of indicated mice (Mean ± S.D.; n = 6 mice per group). (**e**) Representative agarose gel image of genotype PCR products from CD45^+^ splenic cells from indicated mice. (**f**) Graphs showing the disease activity index (DAI; *left panel*), macroscopic score (*middle panel*) and histological score (*right panel*) of indicated mice treated with vehicle or 2% DSS. Individual scoring criteria can be found in Supplementary Tables S1–S3. (**g**) Representative hematoxylin and eosin (H&E) images of colon section from vehicle and DSS treated mice post BMT. (**h**) Representative FACS analysis for CD11b^+^ and F4/80^+^ cells from colons of BMT ANGPTL4^+/+^ and BMT ANGPTL4^−/−^ chimeras treated with vehicle or DSS. n = 6 per group. The Mann-Whitney U test was used. *p < 0.05; **p < 0.01 and ***p < 0.001. n.s. denotes not significant.

To further define the role of epithelial-derived ANGPTL4, we performed bone marrow transplantation (BMT) experiment. We transferred bone marrow from ANGPTL4^+/+^ and ANGPTL4^−/−^ donors to γ-irradiated wild-type ANGPTL4^+/+^ (WT IR) recipient mice (BMT (ANGPTL4^+/+^) or BMT (ANGPTL4^−/−^); Fig. 3a,b). CD4^+^ and CD8^+^ cells were depleted from bone marrow inoculum before BMT to limit possible graft-versus-host-disease (GVHD)-related mortality. The bone marrow of WT IR mice appeared necrotic with a significant reduction in the number of bone marrow cells. The spleen was smaller in size and has reduced CD45^+^ splenic cells (Fig. 3c,d). Hematopoietic reconstitution was observed through the repopulation of bone marrow cells in the femur and CD45^+^ cells in the spleen at 4-week post BMT (Fig. 3c,d). Genotype PCR confirmed the successful reconstitution of the respective donor cells in WT IR mice (Fig. 3e). No significant difference in classical clinical scorings was observed between BMT (ANGPTL4^+/+^) or BMT (ANGPTL4^−/−^) chimeras on vehicle treatment (Fig. 3f). Histological analysis also revealed little difference between BMT (ANGPTL4^+/+^) or BMT (ANGPTL4^−/−^) chimeras (Fig. 3g). As expected, DSS-induced inflammation caused an erosion of the epithelial lining with increased infiltration of immune cells into the lamina propria (Fig. 3g). Notably, there was no significant difference recorded in the DAI, endpoint macroscopic score or histological score between BMT (ANGPTL4^+/+^) and BMT (ANGPTL4^−/−^) chimeras after DSS treatment (Fig. 3f,g and Supplementary Tables S1–3). FACS analysis also showed no significant difference in the number of CD11b^+^ and F4/80^+^ cells in the inflamed colons of BMT (ANGPTL4^+/+^) and BMT (ANGPTL4^−/−^) chimeras (Fig. 3h). Taken together, our data demonstrates the intrinsic role of epithelial-derived ANGPTL4 in regulating colonic leukocyte infiltration during DSS-induced inflammation, and thus the colonic inflammatory landscape.

### ANGPTL4 deficiency alters colonic inflammatory gene expression

**Figure 4:**
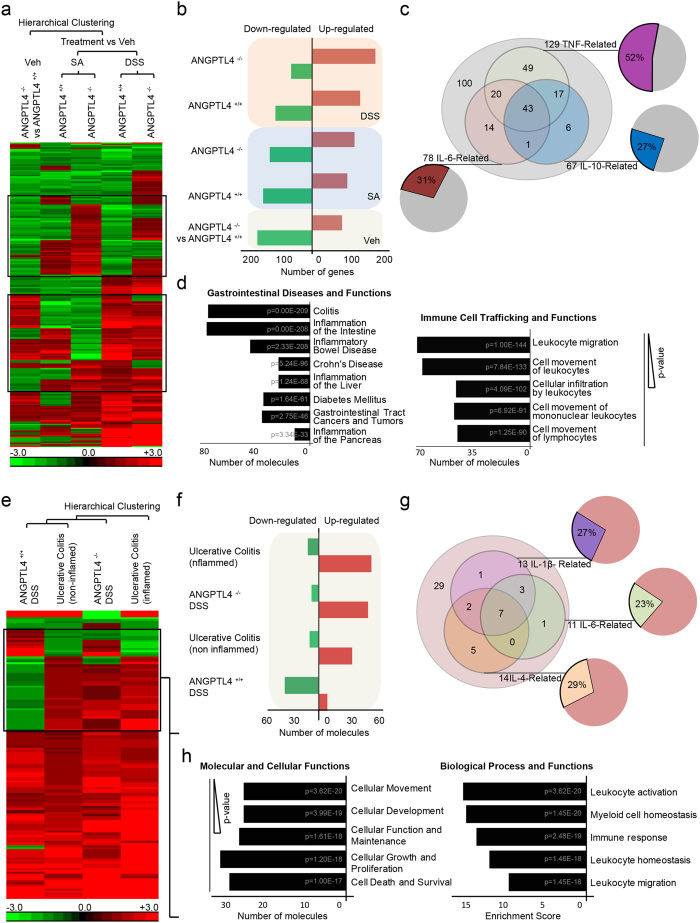
ANGPTL4 deficiency alters colonic inflammatory gene expression. (**a**,**e)** Using Partek Genomic Suite software, we generated a microarray heat map showing changes in gene expression among colon tissues from ANGPTL4^+/+^ and ANGPTL4^−/−^ mice fed with Veh, DSS or SA (**a**) and among colons from ANGPTL4^+/+^ and ANGPTL4^−/−^ mice treated with DSS, inflamed and non-inflamed colon biopsies from ulcerative colitis patients (GSE9452) (**e**). Transcripts were estimated using a log_2_ transformation and subjected to unbiased ANOVA to detect differentially expressed genes between samples. Only genes with a fold change <−1.2 or >1.2 were of significance. The genes were then hierarchical clustered based on significance to generate heatmaps. For (**a**), among the 434 transcripts associated with “gastrointestinal diseases” identified with the IPA database, 63% were found to be commonly regulated across the three treatment groups. For (**e**), 32% of the transcripts associated with “gastrointestinal diseases” were found to be shared among the groups, with the gene profile from DSS-induced inflammation in ANGPTL4^−/−^ mice most closely associated with the inflamed UC biopsies. (**b**,**f)** A larger number of up-regulated genes was correlated with an increasing severity of colonic inflammation in the microarray analysis. **(c)** Gene ontology analysis of gene clusters (black boxes in (**a**)) indicated that the major pathways included IL-6-, IL-10- and TNF-related pathways. **(d)** IPA analysis ranked colitis, inflammation of the intestine and inflammatory bowel disease as the most closely associated gastrointestinal diseases, with leukocyte migration and infiltration as the predominant functions. **(g)** Among the genes that were commonly regulated between human and mouse colon samples (black box), most genes encode for components of the IL-1β, IL-6 and IL-4 pathways. **(h)** The IPA database ranked cellular movement, development, function, growth and proliferation as the top molecular processes involved, along with immune processes such as leukocyte activation, myeloid cell homeostasis and immune responses being the most significant biological functions involved. The numbers in the bar graphs in (**d**,**h)** represent the p-values of the process in the GO analysis, with the smallest p-value being the most significant. An in-depth overview is described in Supplementary Fig. S4.

Our observations suggest that ANGPTL4 deficiency increased the susceptibility to inflammation induced by SA and DSS, albeit with differential severities. To strengthen our findings, we performed a comparative microarray gene expression analysis using colon tissues from ANGPTL4^−/−^ and ANGPTL4^+/+^ mice challenged with either DSS or SA. Using Ingenuity Pathway Analysis (IPA), we identified 250 differentially expressed genes associated with “gastrointestinal diseases”. As expected, the gene expression profiles of DSS-challenged colon samples showed a more aggravated response when compared with the SA-challenged colon samples. Consistent with the above observations, the heat map also indicated that ANGPTL4 deficiency resulted in more severe inflammation in ANGPTL4^−/−^ mice when compared with ANGPTL4^+/+^ mice (Fig. 4a,b and Supplementary Table S4). Further studies of the 2 islands of dissimilarly regulated genes between either challenge groups or genotypes revealed that 52% of the transcripts were related to TNF-α signaling, whereas 27% and 31% were linked to the IL-10 and IL-6 signaling cascades, respectively (Fig. 4c). Gene ontology maps suggested that these genes were primarily involved in leukocyte migration and infiltration (Fig. 4d). The top diseases identified from previous studies listed in the IPA database include colitis, inflammation of the intestine, diabetes mellitus and gastrointestinal tract cancers and tumors (Fig. 4d). Next, a comparative gene expression analysis was performed between DSS-challenged ANGPTL4^−/−^ and ANGPTL4^+/+^ mice and human ulcerative colitis (UC) colonoscopy samples. The colonic mucosal samples from UC patient biopsies were classified as “inflamed” or “non-inflamed”, according to whether the samples exhibited signs of inflammation during collection. Unsupervised hierarchical clustering revealed that the gene expression profile of DSS-challenged ANGPTL4^+/+^ mice was more closely associated with the non-inflamed UC samples, while the profile of ANGPTL4^−/−^ mice was clustered more closely with the inflamed UC samples (Fig. 4e and Supplementary Table S5). Further analysis revealed that the UC samples showed the highest number of up-regulated genes, followed by the DSS-challenged ANGPTL4^−/−^ mice; the DSS-challenged ANGPTL4^+/+^ mice showed the lowest number of up-regulated genes (Fig. 4f). Our analysis also identified a cluster of distinctively regulated genes across both the mouse and human samples. Among these genes, 29%, 27%, and 23%, were involved in the IL-4, IL-1β and IL-6 signaling cascades, respectively (Fig. 4g). Gene ontology analysis revealed that the top molecular and cellular functions included cellular movement, cellular development, cellular function and maintenance, and cellular proliferation, as well as cell death and survival (Fig. 4h). These transcripts have also been found to be involved in myeloid cell homeostasis, immune responses, and leukocyte homeostasis, activation and migration (Fig. 4h). Together, the histological analysis and gene expression profiling indicated that ANGPTL4^−/−^ mice were more susceptible to colonic inflammation, implicating a role for ANGPTL4 in immune cell infiltration.

### ANGPTL4 deficiency increases the infiltration of immune cells

**Figure 5:**
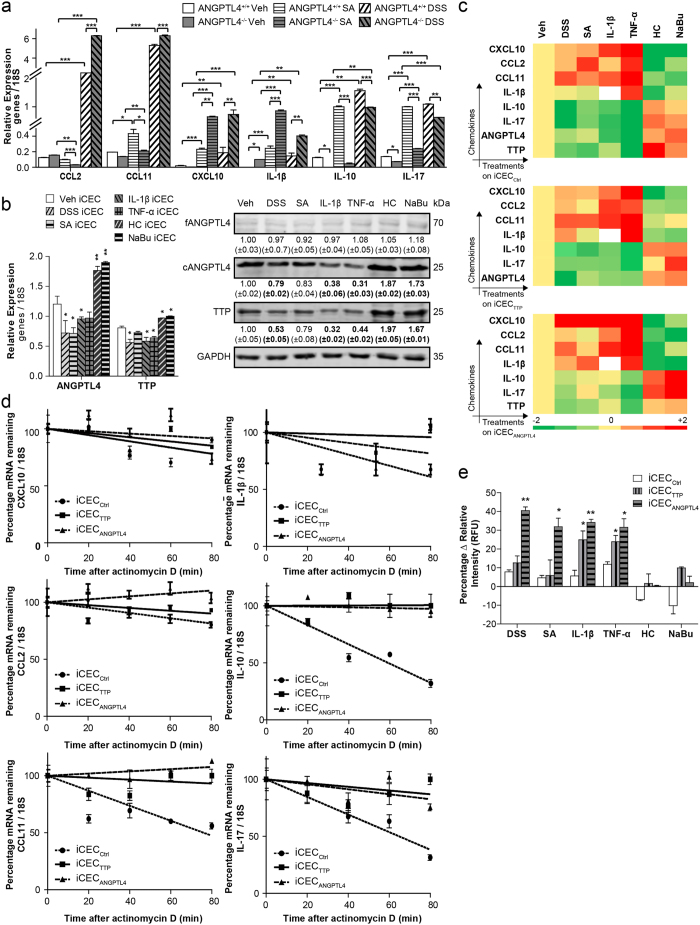
ANGPTL4 deficiency increases the infiltration of immune cells. **(a)** Relative mRNA levels of indicated chemokines in colon tissues from ANGPTL4^+/+^ and ANGPTL4^−/−^ mice treated with Veh, DSS or SA. (**b**) Relative expression (*left panel*) and immunoblot analysis (*right panel*) of ANGPTL4 and TTP levels in iCECs when stimulated with DSS, SA, IL-1β, TNF-α, HC or NaBu. (**c**) Heatmap displaying log-transformed relative expression levels of indicated chemokine mRNA transcripts in iCEC_Ctrl_, iCEC_TTP_ and iCEC_ANGPTL4_ when stimulated with DSS, SA, IL-1β, TNF-α, HC or NaBu. (**d**) Decay curves of IL-1β, IL-10, IL-17, CCL2, CCL11 and CXCL10 in iCEC_ANGPTL4_, iCEC_TTP_ or iCEC_Ctrl_, following actinomycin D treatment after NaBu stimulation. (**e**) Transwell migration assay measuring the percentage change in relative fluorescence intensity of THP1 migration in response to chemokines secreted by iCEC_Ctrl_, iCEC_TTP_ and iCEC_ANGPTL4_ (*bottom panel*). Five independent experiments (n = 5) were performed in triplicate. The Mann–Whitney U test was used. *p < 0.05, **p < 0.01, ***p < 0.001.

ANGPTL4 deficiency exacerbated colonic inflammation upon DSS and SA challenge. Guided by histological differences and gene expression changes between colon samples from ANGPTL4^+/+^ and ANGPTL4^−/−^ mice, we next investigated the chemokine expression profile during inflammation. Consistent with the microarray findings, we detected an overall elevated expression of pro-inflammatory chemokines and a reduced expression of anti-inflammatory cytokines IL-10 and IL-17 in ANGPTL4^−/−^ mice compared with their ANGPTL4^+/+^ littermates (Fig. 5a). The highest levels of these chemokines were also consistent with greater infiltration of inflammatory cells into the colons of ANGPTL4^−/−^ mice (Fig. 1d–f and Supplementary Fig. 1f). The protein concentrations of CCL2, CCL11 and CXCL10 were also found to be elevated in ANGPTL4^−/−^ littermates in both the DSS- and SA- treated groups (Supplementary Fig. S2a). Furthermore, we observe a decreasing trend, albeit not statistically significant, in the protein levels of IL-1β, IL-10 and IL-17 between ANGPTL4^+/+^ and ANGPTL4^−/−^ littermates among different treatments.

To date, little is known about the mechanisms by which ANGPTL4 exerts such effects on the immune and chemokine landscape. Recent publications suggest that ANGPTL4 may modulate the inflammatory response in a variety of disease models^9^,^10^,^11^,^14^,^15^,^23^. Our microarray analysis and a review of the literature directed us to two potential intermediary proteins that respond quickly during acute inflammation and acts broadly to attenuate inflammation^24^,^25^. Human antigen R (HuR) and tristetraprolin (TTP) are mRNA binding proteins well-characterized for their opposite roles in binding mRNA at AU-rich regions located at the 3′-UTR of target transcripts, including chemokines, to modify the inflammatory landscape at the onset of inflammation. HuR is highly selective in its interaction with its cognate mRNA binding partners and has been described as an active participant in promoting mRNA stability^26^,^27^. In contrast, TTP promotes mRNA degradation by destabilizing target transcripts^28^,^29^,^30^. The acute inflammation elicited by DSS and SA treatments significantly suppressed both ANGPTL4 and TTP expression in ANGPTL4^+/+^ littermates (Supplementary Fig. 2b). However, we detected no significant change in HuR expression in both pro-inflammatory treatments. Analysis of our focused gene expression array revealed that the expression of TTP was altered, as was the expression of genes involved in immune cell infiltration and responses. The reduced mRNA and protein levels of TTP in ANGPTL4^+/+^ and ANGPTL4^−/−^ littermates were confirmed by real-time PCR and western blot analyses (Supplementary Fig. 2c). Together, our data suggests the involvement of TTP in the regulation of local colonic inflammation.

To further understand the role that colonic epithelial cells play in regulating chemokine expression, we treated human colon epithelial cells (iCECs) with a panel of pro-inflammatory (DSS, SA, IL-1β or TNF-α) or anti-inflammatory (HC or NaBu) stimuli. Notably, ANGPTL4 and TTP levels decreased when iCECs were treated with DSS, SA, IL-1β and TNF-α (Fig. 5b). Exposure to such pro-inflammatory stimuli increased the levels of CCL2, CCL11, CXC10 and IL-1β mRNA transcripts and decreased the levels of IL-10 and IL-17 mRNA transcripts (Supplementary Fig. 2d). Conversely, anti-inflammatory HC and NaBu increased both ANGPTL4 and TTP levels, prompting a decreased expression of CCL2, CCL11, CXCL10 and IL-1β concomitant with an increase in IL-10 and IL-17 (Fig. 5b and Supplementary Fig. 2d). Next, we examined the role of ANGPTL4 and TTP in mediating the abundance and stability of chemokines. iCECs were subjected to transient ANGPTL4 (iCEC_ANGPTL4_) or TTP knockdowns (iCEC_TTP_) and subsequently exposed to pro- or anti-inflammatory stimulation (Fig. 5c and Supplementary Fig. S2e–g, Tables S6 and 7). As a control, scrambled siRNA was used (iCEC_Ctrl_). Pro-inflammatory stimuli (DSS, SA, IL-1β and TNF-α) increased CCL2, CCL11, CXCL10 and IL-1β expression but decreased IL-10 and IL-17 in iCEC_Ctrl_. Conversely, HC and NaBu stimulation in iCEC_Ctrl_ suppressed CCL2, CCL11, CXCL10 and IL-1β but heightened IL-10 and IL-17. The absence of ANGPTL4 (iCEC_ANGPTL4_) raised the basal levels of CCL2, CCL11, CXCL10, IL-1β, IL-10 and IL-17 expression independent of the nature of stimulation. Interestingly, the CCL11, IL-10 and IL-17 levels surged independent of stimulation when TTP was ablated (iCEC_TTP_). Further investigation into chemokine stability demonstrated that the half-lives of CCL11, IL-10 and IL-17 mRNA transcripts, but not CCL2, IL-1β or CXCL10, were prolonged in iCEC_ANGPTL4_ and iCEC_TTP_ (Fig. 5d). The decreased half-life of TTP in iCEC_ANGPTL4_, and not vice versa, also confirmed that ANGPTL4 positively regulates downstream TTP (Supplementary Fig. S2g). In an effort to understand immune cell infiltration, THP1-derived macrophages were co-cultured with iCECs (iCEC_ANGPTL4_, iCEC_TTP_ and iCEC_Ctrl_) in the presence of various treatments, and macrophage transwell migration was investigated. Consistent with the above results, we found that deficiency in ANGPTL4 and TTP was sufficient to increase chemotactic signals from epithelial cells and accelerate the migration of macrophages (Fig. 5e and Supplementary Fig. S2i). Taken together, our results demonstrate that ANGPTL4 is an important regulator that modulates the chemokine landscape and immune cell infiltration during inflammation. Our data also highlights the role of ANGPTL4 in attenuating inflammation simultaneously through TTP-dependent and independent pathways, although the mechanism for the latter remains to be elucidated.

### ANGPTL4 up-regulates TTP via CREB and NF-κB

**Figure 6:**
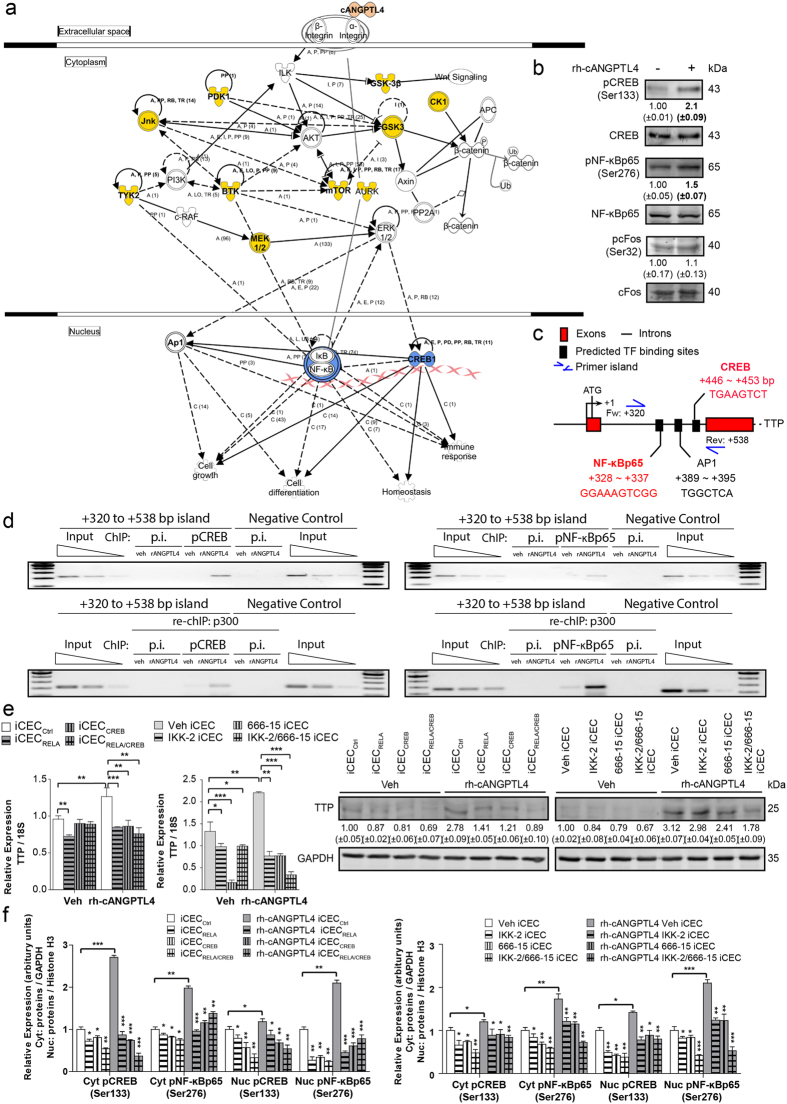
ANGPTL4 up-regulates TTP expression via CREB and NF-κB. (**a)** IPA identified CREB, NF-κB and AP-1 as potential transcription factors that regulate the expression of TTP when stimulated with rh-cANGPTL4. IPA-assisted pathways were mapped following experimental screens using a kinase inhibitor array. **(b)** Immunoblot analysis revealed an increase in the phosphorylation of CREB (pCREB) and NF-κB p65 (pNF-κB) but not cFos (pcFos) after rh-cANGPTL4 stimulation. **(c)** A schematic illustration of the promoter of the human *TTP* gene. The putative transcription factors binding sites were determined *in silico* online with the Jaspar database. The relative location of the ChIP primers was indicated. (**d)** Chromatin Immunoprecipitation (ChIP) and re-ChIP using pCREB or pNF-κB/p65 and coactivator p300 antibodies in iCECs that were stimulated with rh-ANGPTL4. **(e)** Relative expression and immunoblot analysis of TTP for iCECs repressed of CREB and/or NF-κB activity using siRNA (iCEC_Ctrl_, iCEC_RELA,_ iCEC_CREB_ and iCEC_RELA/CREB_) and specific kinase inhibitors (Veh, IKK-2, 666-15 and IKK-2/666-15) in the absence and presence of rh-cANGPTL4. **(f)** Densitometry analysis showing the relative expression of pCREB and pNF-κBp65 in nuclear and cytoplasmic fractions of iCECs as treated in (**e**), normalized against Histone H3 and GAPDH for nuclear and cytoplasmic fractions, respectively, for Supplementary Fig. 3h. The Mann–Whitney U test was used. Three independent experiments (n = 3) were performed.

Full-length ANGPTL4 is proteolytically cleaved into N-terminal coiled-coil (nANGPTL4) and C-terminal fibrinogen-like (cANGPTL4) fragments^31^. To understand which fragment of ANGPTL4 is responsible for regulating TTP expression, we treated iCECs with recombinant (rh) cANGPTL4 and rh-nANGPTL4. This investigation showed that cANGPTL4 was potent in significantly up-regulating TTP expression (Supplementary Fig. 3a). Next, we sought to elucidate the mechanism whereby ANGPTL4 regulates TTP expression. We subjected rh-cANGPTL4-stimulated iCECs to a kinase inhibitor array screen to identify key signaling pathways involved in the cANGPTL4-mediated up-regulation of TTP (Supplementary Fig. 3b). TTP expression was increased when iCECs were treated with rh-cANGPTL4 (Supplementary Fig. 3b, *left panel*). Hence, we reasoned that kinase inhibitors that negated or inhibited the rh-cANGPTL4-mediated up-regulation of TTP mRNA transcripts indicated the involvement of those specific kinases in the selected signaling cascades (*middle panel*). Conversely, kinase inhibitors that did not attenuate the cANGPTL4-mediated up-regulation of TTP would indicate that those kinases were not involved in ANGPTL4-mediated signaling (*right panel*). Our results showed that inhibitors against CDK, JNK, MEK1/2, mTOR, PI3Kδ, TYK2, BTK, CK2, GSK3, PDK1 and Aurora A/B/C targeted key signaling regulators of cANGPTL4-mediated TTP transcription (Supplementary Fig. 3c). To avoid bias, all kinases that prevented the up-regulation of TTP expression under the stimulation of rh-cANGPTL4 were used to study how their availability impacted the overall level of inflammatory signaling. Using IPA, we further established that these signaling mediators resulted in the activation of downstream transcription factors, such as AP1 (cFos-cJun complex), CREB and NF-κB (Fig. 6a). Immunoblot analysis revealed that rh-cANGPTL4 increased the phosphorylation of CREB (pCREB) and NF-κB (pNF-κB) but not phosphorylated cFos (pcFos) (Fig. 6b). *In silico* analysis identified putative CREB (+455 to +453 bp), AP1 (+388 to +395 bp) and NF-κB (+528 to +537 bp) transcription binding sites in the promoter of TTP (Fig. 6c).

To underscore the importance of CREB and NF-κB as active transcription factors mediating TTP expression upon cANGPTL4 stimulation, we inhibited the activity of CREB, NF-κB or both using siRNAs (iCEC_RELA,_ iCEC_CREB_ and iCEC_RELA/CREB_) and specific kinase inhibitors (IKK-2, 666-15 and IKK-2/666-15), respectively. The highly potent CREB inhibitor (666-15) hinders the interaction of KID-KIX, which is essential for CREB-dependent gene transcription^32^, while the IKK-2 inhibitor IV (IKK-2) selectively targets IKK-2 and retards downstream p65NF-kB signaling. In both instances, the lack of either CREB or NF-κB was sufficient to reduce TTP expression in iCECs even in the presence of rh-cANGPTL4 (Fig. 6e and Supplementary Fig. S3g). We also observed reduced pCREB and pNF-κB levels in nuclear and cytoplasmic fractions of iCECs during cANGPTL4-stimulation, suggesting that the nuclear translocation of pCREB and pNF-κB was severely compromised (Fig. 6f and Supplementary Fig. 3h). In conclusion, our data suggests that the CREB and NF-κB transcription factors play irreplaceable roles in the ANGPTL4-mediated, TTP-dependent signaling axis.

## Discussion

The epithelial cells that line the gastrointestinal tract serve as a physiological barrier that prevents the invasion of pathogens and a selective conduit of luminal signals to the host. When harmful immunogens and pathogens are detected, epithelial cells produce chemoattractants to initiate the infiltration of immune cells into the mucosa. Our findings highlight the importance of colonic epithelial cell-secreted ANGPTL4 as a prospective regulator that alters the chemokine landscape in the colon to influence downstream inflammation. We showed that ANGPTL4-deficient mice were more susceptible to acute DSS and SA exposure. 16S metagenomic sequencing indicated little difference in microbe composition between ANGPTL4^+/+^ and ANGPTL4^−/−^ mice that could account for the exacerbated inflammatory response in ANGPTL4^−/−^ mice. Bone marrow transplant study emphasized the intrinsic role of epithelial-derived ANGPTL4 in regulating the inflammatory landscape in the colon. Using human colonic epithelial cells, we further showed that ANGPTL4 regulated the expression of TTP, an mRNA destabilizing agent, via the activation of CREB and NF-κB. The absence of ANGPTL4 or TTP prolonged the mRNA half-life of a specific subset of chemokines.

An exuberant and protracted inflammatory response contributes to the development of many gastrointestinal disorders. Infiltrating macrophages are the major cellular components of this inflammatory response. As the initial cellular barrier, the colonic epithelium plays an important role in the recruitment of inflammatory cells to the mucosa. We showed that ANGPTL4-knockout mice fed DSS or SA exhibited a greater colonic inflammatory response, which was associated with greater infiltration of immune cells when compared with wild-type mice fed similar treatments. Similarly, pro-inflammatory stimuli such as DSS, SA, IL-1β and TNF-α suppressed the expression of ANGPTL4 and TTP in iCECs, whereas anti-inflammatory stimuli such as HC and NaBu increased its expression. In support of these findings, previous studies showed that anti-inflammatory glucocorticoid treatment boosted TTP expression in lymphocytes^33^, while the synthetic glucocorticoid dexamethasone increased TTP levels in A549 cells^34^ as well as ANGPTL4 levels in hepatocytes^35^ and adipocytes^36^. Although the exact pathways remain to be elucidated, dietary NaBu was also found to stimulate ANGPTL4 expression via a PPAR-independent pathway^37^. HC has also been demonstrated to up-regulate IL-10 and IL-17 levels in natural killer cells to improve pneumonia survival rates^38^, although no detailed mechanism was reported. Interestingly, our findings cement the importance of ANGPTL4 in regulating the general inflammatory landscape in the gastrointestinal tract. Although other ANGPTL4-dependent but TTP-independent signaling mechanisms remain to be identified, we demonstrated that ANGPTL4 modifies the availability of a subset of chemokine signals that alter the ease of immune cell infiltration through a TTP-dependent signaling circuit.

Confluent iCECs mimic an intact gastrointestinal mucosal layer and express low basal levels of ANGPTL4 and TTP. Many previous studies have utilized cancerous lines, such as HT-29, Caco-2 and HCT-116 cells, as surrogates for an *in vitro* gastrointestinal model^39^,^40^. Potentially, this ANGPTL4-dependent mechanism may be impaired or altered in cancerous lines, as the expression of ANGPTL4 is known to be elevated in many types of cancer^7^. We further established that iCECs secreted ANGPTL4, which acted in an autocrine manner to induce the expression of TTP. We previously showed that secreted ANGPTL4 bound and activated integrin-mediated pathways^41^. Indeed, TTP is an important factor that contributes to mediating, modulating and attenuating inflammatory responses. Using the combination of an unbiased kinase inhibitor screen assay, along with ChIP and re-ChIP experiments, our current work unveiled multiple signaling conduits whereby ANGPTL4 stimulates TTP expression, resulting in CREB and NF-κB activation. Interestingly, BTK and Aurora inhibitors were identified to significantly inhibit the ANGPTL4-dependent up-regulation of TTP, suggesting that they exert dominant roles in this signaling cascade. Lending support for a role for Aurora in NF-kB activation, Katsha A *et al*. also reported that Aurora kinase A promoted and sustained inflammation through NF-κB, leading to tumor formation^42^. Again, the importance of Aurora in the ANGPTL4-mediated up-regulation of TTP may be compromised when studied in cancer cell lines. Our study also emphasizes a potential role for Aurora kinases in regulating inflammation. Similarly, other studies have shown that cancer cells that express low levels of TTP are correlated with a genetic signature of low expression of CREB-related target genes^43^. Numerous studies have also shown that TTP binds to AU-rich sequences at the 3′-UTRs of specific mRNA transcripts such as TNF-α^44^, IFN-γ^45^, IL-10 and IL-17^46^. In agreement with these previous reports, we showed that the mRNA half-lives of specific inflammatory chemokines were prolonged in the absence of ANGPTL4 and TTP.

The pathogenesis of inflammatory bowel disease in humans is complex and has a multifactorial and diverse aetiology^47^. Dysbiosis has been associated with inflammation and impaired mucosal immune function in intestine of humans^48^,^49^,^50^. Our 16S sequencing revealed little difference in microbe composition between two genotypes that can account for the exacerbated colonic inflammatory response in ANGPTL4^−/−^ mice. In this respect, the extent to which ANGPTL4 expression defines the colonic microbiota profiles remains to be delineated. It is also conceivable that mechanisms other than ANGPTL4-TTP-mediated chemokine mRNA stability might be involved. Our microarray analyses showed that Lrg1 and Gpx2 expression was up-regulated by 6- and 4-fold, respectively, in the colons of ANGPTL4^−/−^ mice compared with wild-type counterparts. Lrg1 is proposed to be a possible biomarker for the diagnosis of ulcerative colitis^51^. Both Lrg1 and Gpx2 proteins are up-regulated during the acute stages of pediatric appendicitis^52^ and colitis^53^, respectively. These data suggest that additional ANGPTL4-dependent mechanisms may contribute to the severity of colitis in ANGPTL4^−/−^ mice. Furthermore, ANGPTL4 has been implicated in many inflammation-associated pathologies. Although not explored in the present study, the mechanism whereby ANGPTL4 may be targeted to influence cell infiltration via TTP-mediated chemokine mRNA stability could be applicable to these pathologies.

## Materials and Methods

### Antibodies and Reagents

Antibodies against c-Fos (#2250), phospho-c-Fos (Ser32; #5348), Elk-1 (#9182), phospho-Elk-1 (Ser383; #9186), CREB (#48H2) and phospho-CREB (Ser133; #9198) were from Cell Signaling (USA). Antibodies against Histone H3 (05-499), NF-κB/p65 (MAB3026) and phospho-NF-κB/p65 (Ser276; AB3375) were from Merck Millipore, USA. Antibodies against Ly-6G (#127602 and #127614), CD68 (#137002) were from Biolegend, USA. FACS antibodies (APC-/FITC-conjugated) against CD11b, CD45, F4/80 were from Miltenyi Biotec, USA. Anti-CD4- and anti-CD8a- conjugated microbeads (#130-049-201, #130-049-401) was purchased from Miltenyi Biotec, USA. Anti-TTP (sc-14030, Santa Cruz, USA), active/cleaved Caspase 3 (NB100-56113, Novus Biological, USA),

ChIP-grade antibodies against NF-κB/p65 (ab7970) and CREB (ab31387) were from Abcam, UK and p300 (RW128) from Upstate Biotechnology, USA. IRDye^®^ 680 LT goat anti-rabbit IgG, goat anti-mouse IgG and donkey anti-goat IgG were purchased from Li-Cor Biosciences, USA. Dextran sulfate sodium salt (DSS; 36 000 to 50 000M.Wt) was from Affymetrix, USA (#14489). Recombinant human cANGPTL4 (rh-cANGPTL4) and anti-ANGPTL4 antibodies were produced in-house as previously described^24^. Recombinant human nANGPTL4 (rh-nANGPTL4; 8249-AN, RnD Systems), IL-1β (ab9617, Abcam), TNF-α (#717904, BioLegend) and hydrocortisone (CAS 50-23-7, Calbiochem). All other chemicals were purchased from Sigma–Aldrich.

### Animals

ANGPTL4^+/−^ C57BL/6J mice were acquired from the Mutant Mouse Regional Resource Center (MMRRC) and were generated by Genentech. The mice were crossed to produce ANGPTL4^+/+^ and ANGPTL4^−/−^ offspring. DNA from mouse ear clippings was isolated using the KAPA Express Extract reactions and genotyped using Mouse Genotyping HotStart in accordance with the manufacturer’s recommendations (KAPA Biosystems, USA).

The PCR products were visualized on 2% agarose gels stained with SYBR Safe (ThermoFisher Scientific, USA). In the DSS treatment study, age-matched ANGPTL4^+/+^ and ANGPTL4^−/−^ males (n = 20) were treated with 5% DSS in the drinking water for 8 days; the DSS was changed every 2 days. In the SA study, mice (n = 20) were fed with a 15% SA:85% ground chow (w/w) diet for 8 days. The disease activity index (DAI) was assessed every alternate day, while macroscopic and histological scorings were performed at the experimental endpoint. The scoring criteria are available in Supplementary Tables S1–3. Protein and RNA samples were collected by scraping colonic epithelial cells. Animal experiments were approved by and carried out in accordance with the guidelines of Nanyang Technological University’s Institutional Animal Care and Use Committee (NTU, IACUC, ARF SBS/NIE-A0321 and ARF SBS/NIE-A0324), Singapore.

### Cell Culture

SV40**-**immortalized human colon epithelial cells (iCECs; T0570; ABM Canada) were cultured in Prigrow III Medium (ABM Canada) in collagen-coated flasks and maintained at 5% CO_2_ at 37 °C. Confluent iCECs were stimulated with either 1 μg/mL DSS, 500 μM SA, 10 ng/mL IL-1β, 10 ng/mL TNF-α, 0.4 ug/mL HC or 2 mM NaBu for 6 h prior to harvesting. The siRNA knockdown against ANGPTL4, CREB, RELA or TTP was accomplished using Dharmacon ON-TARGETplus siRNA (Supplementary Table S8) according to the manufacturer’s protocol (Thermo Scientific, USA). Inhibition of the transcription factors CREB and IKK were carried out using 0.1 μM CREB inhibitor (666-15; #5661; Tocris Bioscience, UK) and/or 1 μM IKK-2 inhibitor IV (IKK-2; 40-1481; Merck Millipore) respectively. iCEC_ANGPTL4_, iCEC_TTP_ and iCEC_Ctrl_ were pre-treated with 2 mM NaBu for 2 h, followed by10 μg/mL actinomycin D for half-life measurements. *In vitro* experiments were carried out in triplicate.

### RNA Extraction and Real-time Quantitative PCR

RNA was extracted using TRIzol (Invitrogen, USA) and was reverse transcribed using the iScript^TM^ Reverse Transcription Supermix according to the manufacturer’s recommendations (Bio-Rad, USA). Quantitative PCR (qPCR) was conducted as previously described^54^. Primer sequences are presented in Supplementary Table S9. The relative expression levels of the respective mRNAs described in this manuscript were normalized against those of the housekeeping ribosomal 18S RNA.

### Microarray

RNA was transcribed into cDNA using an Applause WT-Amp ST System in accordance with the manufacturer’s recommendations (NuGEN Technologies, USA). cDNA was purified using a MinElute Reaction Cleanup Kit (Qiagen, USA), fragmented and labeled using an Encore Biotin Module kit (NuGEN Technologies, USA). Labeled cDNA molecules were hybridized using the Affymetrix hybridization master mix and injected onto GeneChip Mouse Gene 1.0 ST Array gene chips (Affymetrix, USA) for scanning. Partek Genomic Suite (Partek Inc., USA) and Ingenuity Pathway Analysis (IPA) software were used to perform analyses, an overview of which is available in Supplementary Fig. S4. The human colonic mucosal samples used for the comparative analyses in Fig. 3e–h were published by Olsen *et al*. under the GEO accession number GSE9452^55^. The microarray data (accession number GSE78500) have been deposited in the GEO database.

### Protein Extraction and Western Blot Analysis

Proteins were extracted in ice-cold lysis buffer (20 mM Na_2_H_2_PO_4_, 250 mM NaCl, 1% Triton-X, 0.1% SDS, 1 mM PMSF, and 200 μM sodium orthovanadate) as previously described^44^. Equal amounts of proteins were resolved using SDS-PAGE gels and electro-transferred to low-fluorescence PVDF membranes (IPFL00010; Merck Millipore, USA). The cytoplasmic and nuclear fractions were isolated using the NE-PER Nuclear and Cytoplasmic Extraction Reagents (#78833; Thermo Scientific). Briefly, all protein samples for western blot analysis performed in this manuscript were first normalized using the NanoDrop 3300 Spectrophotometer (Thermo Scientific) prior to the ensuing downstream analyses. All loading controls for immunoblot analyses were obtained from the same sample. Membranes were blocked with 0.5 × Odyssey Blocking Buffer (LI-COR Biotechnology, USA) and probed with the respective primary antibodies overnight at 4 °C in 1× Blocking Buffer with 0.1% Tween-20. Membranes were washed thrice in TBST (50 mM Tris HCl, pH 7.6, 150 mM NaCl, 0.1% Tween-20) for 5 min each and incubated for 1 h with the respective secondary antibodies in 1× Blocking Buffer at room temperature. Each membrane was washed thrice in TBST and then air-dried. Images were analyzed using a CLx scanner and Image Studio V2.1 (LI-COR Biosciences, USA). All membranes were first probed for target proteins before being re-probed for GAPDH housekeeping protein. Densitometry analyses were processed using ImageJ (NIH, USA). Densitometry values are presented under each blot, with values in bold representing significance (p < 0.05), or in graphs (for Fig. 4e and Supplementary Fig. 3h). The LEGENDplex Multi-Analyte Flow Assay Kit was used to measure the protein concentrations of targets (CCL2, CCL11, CXCL10, IL-1β, IL-10 and IL-17) for both mouse and humans and was customized through Biolegend, USA and performed in accordance with the manufacturer’s recommendations.

### Tissue Processing, H&E, Immunofluorescence and TUNEL Staining

Tissues were harvested and fixed in 4% paraformaldehyde overnight at 4 °C and subsequently were dehydrated and embedded in paraffin as previously described^56^. Tissues were rehydrated and stained with hematoxylin and eosin (H&E) according to the manufacturer’s protocol (Sigma–Aldrich, USA). Slides were mounted with Fluka Eukitt quick-hardening mounting medium (Sigma–Aldrich, USA). Images were obtained using a Zeiss Axiovert 200M microscope with a Zeiss FLUAR 10x/0.5 NA objective and PALMRobo V4.3 software (Carl Zeiss, Germany).

The TUNEL assay was performed in accordance with the manufacturer’s protocol (Roche). For immunofluorescence analyses, tissue sections were rehydrated and incubated in 5% normal goat serum (NGS) for 1 h and then incubated with the primary antibodies in 3% NGS overnight at 4 °C. Subsequently, sections were washed and then incubated with respective secondary Alexa Fluor 594-conjugated antibodies (Invitrogen) for 1 hour at room temperature. Slides were then mounted with Hoechst 33342 dye (Life Technologies, USA). Images were obtained using a LSM 710 confocal microscope with a Zeiss EC Plan-NEOFLUAR 20x/0.5 NA objective and were analyzed using ZEN 2012 Blue Edition software (Carl Zeiss, Germany).

### Tissue Dissociation and Flow Cytometry

Mouse colon tissues from various treatment groups were first homogenized into single-cell suspensions using the gentleMACS Dissociator (Miltenyi Biotec, USA) in DMEM containing 1 mg/mL collagenase-3 and 40 units/mL DNase 1. Homogenates were filtered through a 70 μm nylon cell strainer, washed and finally resuspended in PBS blocking buffer containing 3% FBS. The resultant suspension was then incubated with the respective FITC-/APC- conjugated antibodies on ice. Samples were washed twice and resuspended in PBS, and were then subjected to FACS analysis on the Accuri C6 Flow Cytometer (BD Biosciences, USA). All FACS analyses were performed for 5000 events.

### Transwell Migration Assay

THP1 monocytes were differentiated in 100 ng/mL TPA complete medium for 48 h and allowed to recover for 24 h on transwell inserts. Respective siRNA knockdowns were performed on iCECS (iCEC_ANGPTL4_, iCEC_TTP_ and iCEC_Ctrl_). Inserts containing differentiated THP1 cells were then introduced to the transfected iCECs and exposed to various pro- and anti- inflammatory stimuli for 10 h (Fig. 3e). Inserts were then washed with PBS twice and fixed in 1% glutaraldehyde for 10 min, rinsed with PBS and stained with SYTO 60 (Thermo Fisher, USA) for 30 min. Cotton buds were used to remove all unmigrated cells trapped in the upper chamber of the inserts. Inserts were rinsed again in PBS before the quantification of fluorescence using the CLx scanner and Image Studio V2.1 (LI-COR Biosciences, USA). Relative fluorescence as a readout for cellular migration was calculated by normalizing the intensities between test wells (THP1 and iCECs) and control wells (THP1 without iCECs).

### Chromatin Immunoprecipitation (ChIP) and Re-ChIP

ChIP and re-ChIP experiments were performed as described previously^57^. In addition, samples were subjected to washes with increased stringencies: twice each with the low (1% Triton X-100, 0.1% SDS, 2 nM EDTA, 20 mM Tris-HCl, 150 mM NaCl at pH 8.1) and high salt (1% Triton X-100, 0.1% SDS, 2 nM EDTA, 20 mM Tris-HCl, 500 mM NaCl at pH 8.1) wash buffers. Primer sequences are presented in Supplementary Table S9.

### Kinase Inhibitor Array

Immortalized human colon cells were subjected to treatment with 95 kinase inhibitors (SYN-2103; SYNkinase, Australia) in the presence or absence of rh-c-ANGPTL4 (8 μg/mL) for 6 h (Supplementary Table S10). RNA was then isolated and reverse transcribed as described above.

### 16S Metagenomics Sequencing

Post-weaned, age- and gender-matched ANGPTL4^−/−^ or ANGPTL4^+/+^ mice were used. Fresh fecal pellets were collected before and after DSS treatment. Bacterial genomic DNA was isolated from feces using the FASTDNA spin kit (#116570200, MP Biomedicals, USA). 16S sequencing of the 16S V4 variable region was performed by SeqMatic LLC, USA using the Illumina MiSeq sequencing platform.

### Bone Marrow Transplant Model

BMT was performed using bone marrow cells isolated from donor mice (ANGPTL4^−/−^ or ANGPTL4^+/+^) and transplanted into recipient γ-irradiated ANGPTL4^+/+^ wild-type mice (WT IR; n = 6 per group). Recipient WT mice were kept on acidified water (pH 3.0) over the course of the BMT. WT mice were medicated with sulfamethoxazole (40 mg/kg) and trimethoprim (8 mg/kg) in oral suspension for one week prior to and after γ-irradiation (9.5 Gy) using the Biobeam 8000 (Gamma-Service Medical GmbH, Germany). Red blood cells from donor bone marrow cells were lyzed (0.89% NH_4_Cl, 0.1 mM EDTA, pH 7.2). Residual bone marrow cells were washed with PBS and filtered through 30 μM nylon cell strainer before depleting CD4^+^ and CD8^+^ cell populations using the QuadroMACS kit (#130-091-051, Miltenyi Biotec, USA). Approximately 10^7^ CD4^+^ and CD8^+^ depleted bone marrow cells were introduced to WT IR mice via retro-orbital injection. Chimera mice [BMT (ANGPTL4^+/+^) and BMT (ANGPTL4^−/−^)] were allowed to recover for 4-weeks before 2% DSS treatment for 8 days. Bone marrow reconstitution was determined using genotype PCR (Supplementary Table S11). PCR products were visualized on 2% agarose gels tinted with SYBR Safe (ThermoFisher Scientific, USA). Cell count was numerated using the automated ADAM-MC cell counter (NanoEntek, USA). Cells from the spleen and colon were analyzed for immune cell infiltration using FACS (BD LSRFortessa X-20).

### Statistical Analysis

Statistical analyses were performed using two-tailed Mann-Whitney U tests. P-values < 0.05 denote statistically significant differences between means; *p < 0.05; **p < 0.01 and ***p < 0.001. Values are expressed as the means ± standard error.

## Additional Information

**How to cite this article:** Phua, T. *et al*. Angiopoietin-like 4 Mediates Colonic Inflammation by Regulating Chemokine Transcript Stability via Tristetraprolin. *Sci. Rep.* **7**, 44351; doi: 10.1038/srep44351 (2017).

**Publisher's note:** Springer Nature remains neutral with regard to jurisdictional claims in published maps and institutional affiliations.

## Supplementary Material

Supplementary File

Supplementary Tables
