# Supplementary material for: Angiopoietin-like 4 Mediates Colonic Inflammation by Regulating Chemokine Transcript Stability via Tristetraprolin
Source: Sci Rep. 2017 Mar 13;7:44351. doi: 10.1038/srep44351 (PMC5347094; doi:10.1038/srep44351)
Supplement: Supplementary File [file srep44351-s1.docx]

**SUPPLEMENTAL INFORMATION**

**Angiopoietin-like 4 Mediates Colonic Inflammation by Regulating Chemokine Transcript Stability via Tristetraprolin.**

Terri PHUA^1,2^, Ming Keat SNG^1,3^, Eddie Han Pin TAN^1^, Dickson Shao Liang CHEE^1^, Yinliang LI^1^, Jonathan Wei Kiat WEE^1^, Ziqiang TEO^1^, Jeremy Soon Kiat CHAN^1^, Maegan Miang Kee LIM^1^, Chek Kun TAN^3^, Pengcheng ZHU^1^, Velmurugesan ARULAMPALAM^2^, Nguan Soon TAN^1,3, 4,5*^

^1^School of Biological Sciences, Nanyang Technological University, 60 Nanyang Drive, Singapore 637551

^2^Department of Microbiology, Tumor and Cell Biology, Karolinska Institutet, Nobels vӓg 16, Stockholm 17177, Sweden

^3^Lee Kong Chian School of Medicine, Nanyang Technological University, 50 Nanyang Drive, Singapore 639798

^4^Institute of Molecular Cell Biology, 61 Biopolis Drive, Proteos, Agency for Science Technology & Research, Singapore 138673

^5^KK Research Centre, KK Women’s and Children Hospital, 100 Bukit Timah Road, Singapore 229899

**Correspondence to NST:** [nstan@ntu.edu.sg](mailto:nstan@ntu.edu.sg); Tel: +65 6316 2941; Fax: +65 6791 3856.

**Supplementary Table S1.** Disease Activity Index (DAI) Score.

| Score | Weight Loss | Stool Consistency | Visible Blood in Feces |
| --- | --- | --- | --- |
| 0 | 0% | None | None |
| 1 | 1-5% |  |  |
| 2 | 5-10% | Loose Stool | Slight Bleeding |
| 3 | 10-20% |  |  |
| 4 | >20% | Diarrhoea | Extensive Bleeding |

**Supplementary Table S2.** Endpoint Macroscopic/Disease Severity Score.

| Score | Rectal Bleeding | Rectal Prolapse | Stool Consistency | Blood |
| --- | --- | --- | --- | --- |
| 0 | None | None | Normal | Normal |
| 1 | Red | Signs of prolapse | Soft | Red |
| 2 | Dark Red | Clear prolapse | Very Soft | Dark Red |
| 3 | Gross Bleeding | Extensive Prolapse | Diarrhea | Black |

**Supplementary Table S3.** Histological Scoring for Degree of Epithelial Damage and Inflammatory Infiltration.

| Score | Inflammation Severity | Crypt Damage | Ulceration |
| --- | --- | --- | --- |
| 0 | Rare inflammatory cells in the lamina propria | Intact crypts | absence of ulcer |
| 1 | Increased numbers of granulocytes in the lamina propria | Loss of the basal one-third | <2 foci of ulcerations |
| 2 | Confluence of inflammatory cells extending into the submucosa | Loss of the basal two-thirds | < 4 foci of ulcerations |
| 3 | Transmural extension of the inflammatory infiltrate | Loss of entire crypts | confluent or extensive ulceration |
| 4 | - | Change of epithelial surface with erosion | - |
| 5 | - | Confluent erosion | - |

Please refer to the respective tabs in the attached Excel file for the datasets in **Supplementary Tables S4-7** and **S10.**

**Supplementary Table S4.** List of Differentially Expressed Genes and their respective expression values in association with Gastrointestinal Diseases.

**Supplementary Table S5.** List of Differentially Expressed Genes and their respective expression values in association with Gastrointestinal Diseases in comparision between murine and human samples.

**Supplementary Table S6.** Fold change of chemokine expression in iCECs when treated with various pro- and anti-inflammatory stimuli.

**Supplementary Table S7.** Fold change of chemokine concentration in iCECs when treated with various pro- and anti-inflammatory stimuli.

**Supplementary Table S10.** Concentrations of kinase inhibitors in the Kinase Inhibitor Array used in this study.

**Supplementary Table S8.** Sequences of human ON-TARGETplus SMARTPool siRNA targets for ANGPTL4-, CREB-, RELA- or TTP-knockdown.

| **Gene Symbol** | **Species** | **siRNA smartpool target sequences (5' to 3')** |
| --- | --- | --- |
| **ANGPTL4** | Human | GAUGGAGGCUGGACAGUAA |
|  |  | CCACUUGGGACCAGGAUCA |
|  |  | GAAAGAGGCUGCCCGAGAU |
|  |  | GGCAGAAGCUUAAGAAGGG |
| **CREB1** | Human | GAGAGAGGUCCGUCUAAUG |
|  |  | UAGUACAGCUGCCCAAUGG |
|  |  | CAACUCCAAUUUACCAAAC |
|  |  | GCCCAGCCAUCAGUUAUUC |
| **RELA** | Human | GGAUUGAGGAGAAACGUAA |
|  |  | CCCACGAGCUUGUAGGAAA |
|  |  | GGCUAUAACUCGCCUAGUG |
|  |  | CCACACAACUGAGCCCAUG |
| **TTP** | Human | GCAAGUAGCCAAAGCCGUU |
|  |  | CCCAAAUACAAGACGGAAC |
|  |  | GGACAGUAAUCAAGUAAUC |
|  |  | GCAUAUUUAAGGGAGGCAA |

**Supplementary Table S9.** Sequences of real-time PCR primers used in this study.

| **Gene Symbol** | **Species** | **Gene ID** | **Forward primer sequence**  **(5' to 3')** | **Reverse primer sequence**  **(5' to 3')** |
| --- | --- | --- | --- | --- |
| 18S | Human/  Mouse | 19791 | GTAACCCGTTGAACCCCATT | CCATCCAATCGGTAGTAGCG |
| ANGPTL4 | Human | 51129 | TGGTTTGGCACCTGCAGCCATTC | TGCTGCCATGGGCTGGATCAAC |
| ANGPTL4 | Mouse | 57875 | CCCCACGCACCTAGACAATG | GCCTCCATCTGAAGTCATCTCA |
| CCL2 | Human | 6347 | CAGCCAGATGCAATCAATGCC | TGGAATCCTGAACCCACTTCT |
| CCL2 | Mouse | 20296 | TTAAAAACCTGGATCGGAACCAA | GCATTAGCTTCAGATTTACGGGT |
| CCL11 | Human | 6356 | CCCCTTCAGCGACTAGAGAG | TCTTGGGGTCGGCACAGAT |
| CCL11 | Mouse | 20292 | GAATCACCAACAACAGATGCAC | ATCCTGGACCCACTTCTTCTT |
| CREB1 | Human | 1385 | TTAACCATGACCAATGCAGCA | TGGTATGTTTGTACGTCTCCAGA |
| CXCL10 | Human | 3627 | GTGGCATTCAAGGAGTACCTC | TGATGGCCTTCGATTCTGGATT |
| CXCL10 | Mouse | 15945 | CCAAGTGCTGCCGTCATTTTC | GGCTCGCAGGGATGATTTCAA |
| IL-1β | Human | 3553 | ATGATGGCTTATTACAGTGGCAA | GTCGGAGATTCGTAGCTGGA |
| IL-1β | Mouse | 16176 | GAAATGCCACCTTTTGACAGTG | TGGATGCTCTCATCAGGACAG |
| IL-6 | Mouse | 16193 | TCTATACCACTTCACAAGTCGGA | GAATTGCCATTGCACAACTCTTT |
| IL-10 | Human | 3586 | GACTTTAAGGGTTACCTGGGTTG | TCACATGCGCCTTGATGTCTG |
| IL-10 | Mouse | 16153 | CTTACTGACTGGCATGAGGATCA | GCAGCTCTAGGAGCATGTGG |
| IL-17 | Human | 3605 | TCCCACGAAATCCAGGATGC | GGATGTTCAGGTTGACCATCAC |
| IL-17 | Mouse | 16171 | CGGAGAATTAGTCCCTGTGTTG | GAACAGTCACTTCATACTCCTGG |
| IFN-γ | Mouse | 15978 | ACAGCAAGGCGAAAAAGGATG | TGGTGGACCACTCGGATGA |
| pCREB/pNF-κB Binding Site on TTP | Human | - | TGCAAGTGGAAAGTCGGAG | TGGAAGTCCGGATTGCTTC |
| pCREB/pNF-κB Negative Control on TTP | Human | - | CCAGACAGAGGGAACTGCAAG | GAGCATGCAGTGTGCACCAG |
| RELA | Human | 5970 | GTGGGGACTACGACCTGAATG | GGGGCACGATTGTCAAAGATG |
| TTP | Human | 7538 | GACTGAGCTATGTCGGACCTT | GAGTTCCGTCTTGTATTTGGGG |
| TTP | Mouse | 22695 | CCGAATCCCTCGGAGGACTT | GAGCCAAAGGTGCAAAACCA |
| TNF-α | Mouse | 21926 | CCCTCACACTCAGATCATCTTC | GCTACGACGTGGGCTACAG |

**Supplementary Table S11.** Genotyping PCR primer sequences used in this study.

| **Gene** | **Species** | **Forward primer sequence**  **(5' to 3')** | **Reverse primer sequence**  **(5' to 3')** |
| --- | --- | --- | --- |
| ANGPTL4^+/+^ | Mouse | GCAAGATCCAGCAATTGTTCCAG | ACCCTTGATGAGAGCCTAGTGAG |
| ANGPTL4^-/-^ | Mouse | GCAAGATCCAGCAATTGTTCCAG | GGTGCCCCAAGGACCTGAAATGA |

**Supplementary Figures and Legends**

**
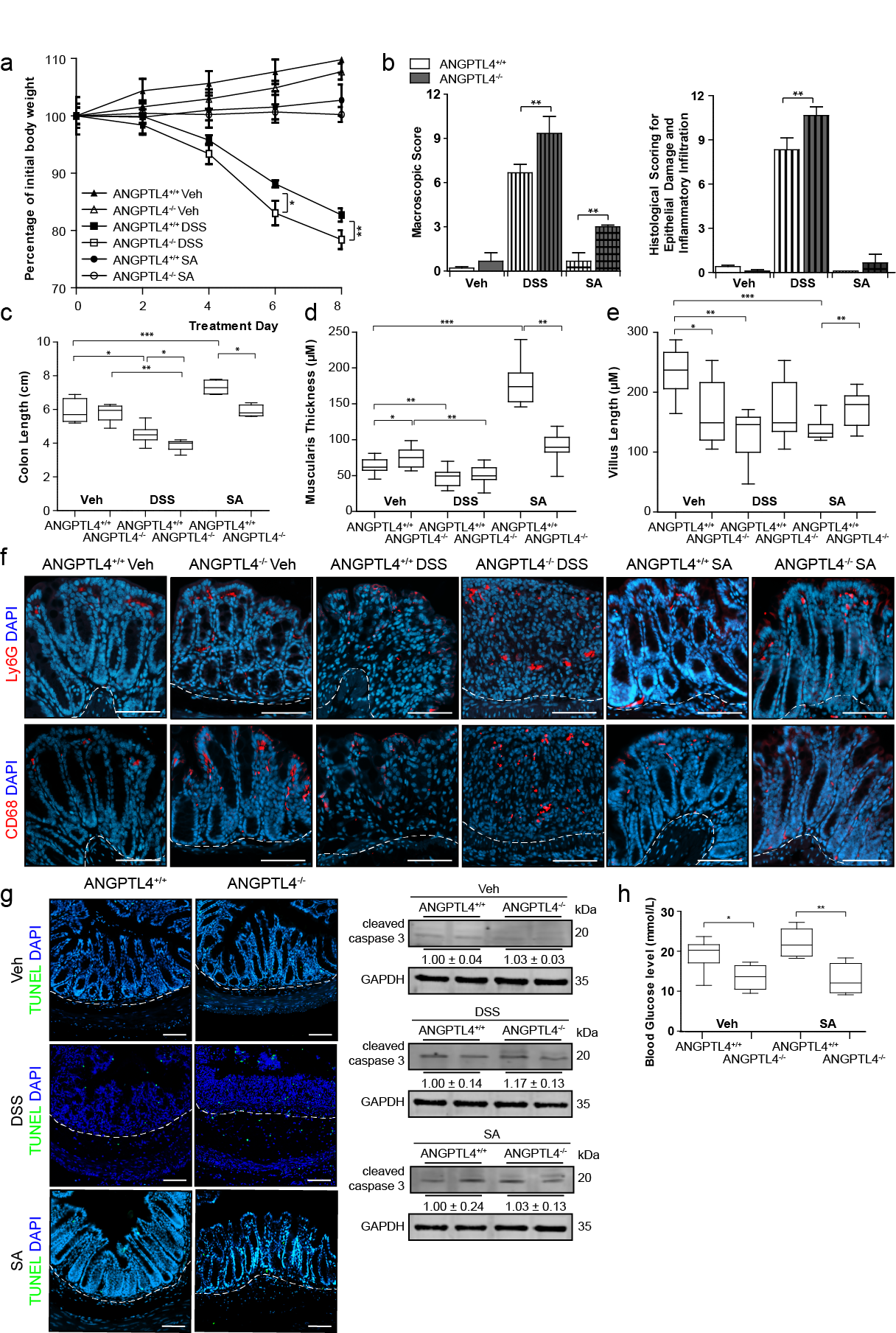
**

**Supplementary Fig. S1. Parameters of colon samples from ANGPTL4^+/+^ and ANGPTL4^-/-^ mice given Veh, DSS and SA at 8-day post-treatment.**

**(a)** Percentage change in body weight of ANGPTL4^+/+^ and ANGPTL4^-/-^ mice given Veh, DSS or SA over an 8-day treatment. (**b**) Macroscopic (*left panel*) and histological (*right panel*) scoring for mice at the endpoint; scoring criteria can be found in Supplementary Tables 2-3 respectively. Quantitative measurements of **(c)** colon length, **(d)** muscularis thickness and **(e)** villus length at the 8-day post-treatment experimental endpoint. **(f)** Immunofluorescence staining for Ly6G (neutrophils; red) and CD68 (macrophages; red) and nuclei (blue) in colon samples from ANGPTL4^+/+^ and ANGPTL4^-/-^ mice following the indicated treatments. **(g)** TUNEL staining (*left panel*) and immunoblot analysis of cleaved caspase 3 (*right panel*) and for apoptotic cells in colon sections from ANGPTL4^+/+^ and ANGPTL4^-/-^ mice following the indicated treatment. Scale bar = 100 µm. **(h)** Quantitative measurements of plasma blood glucose levels in ANGPTL4^+/+^ and ANGPTL4^-/-^ mice after Veh, DSS and SA treatments for 8 days. For the Veh, DSS or SA groups, n=20 mice were used in each treatments. The Mann–Whitney U test was used. *p < 0.05, **p < 0.01, ***p < 0.001.


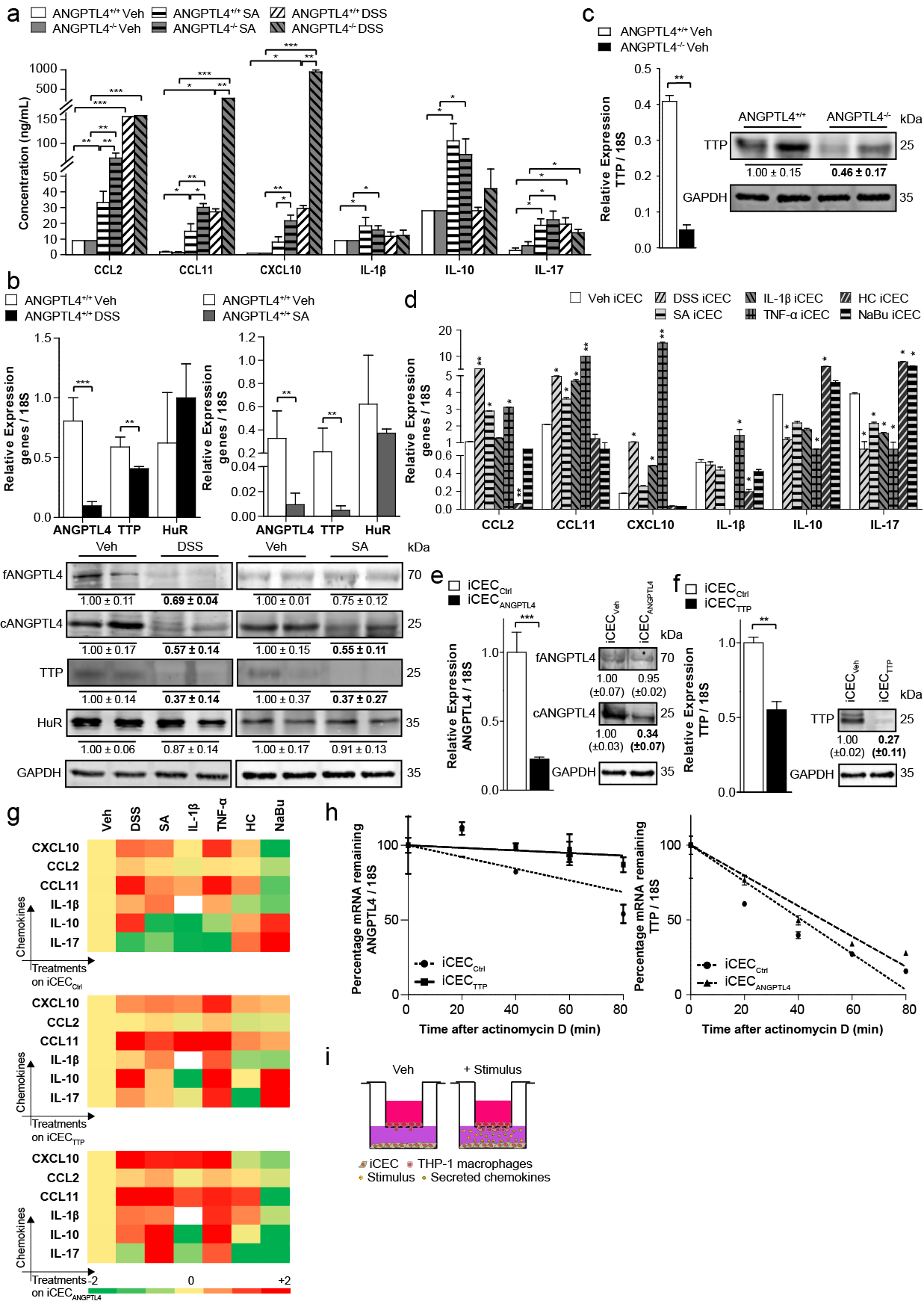


**Supplementary Fig. S2. Chemokine expression levels in ANGPTL4^+/+^ and ANGPTL4^-/-^ mice.**

**(a)** Relative concentration of the indicated chemokines in colon tissues from ANGPTL4^+/+^ and ANGPTL4^-/-^ mice treated with Veh, DSS or SA. (**b**) Relative expression (*upper panel*) and immunoblot analysis (*lower panel*) of ANGPTL4, TTP and HuR levels in ANGPTL4^+/+^ littermates for DSS and SA treatments with respect to Veh. (**c**) Relative mRNA and protein levels of TTP in colon tissues from ANGPTL4^+/+^ and ANGPTL4^-/-^ mice. **(d)** Relative mRNA expression of the indicated chemokines in iCECs treated with DSS, SA, IL-1β, TNF-α, HC or NaBu. Relative mRNA (*left panel*) and protein (*right panel*) levels accounting for levels of (**e**) ANGPTL4 and **(f)** TTP following siRNA knockdown as determined by real-time PCR and immunoblot analysis, respectively. (**g**) Heatmap showing log-transformed protein concentration of indicated chemokine mRNA transcripts in iCEC_Ctrl_, iCEC_TTP_ and iCEC_ANGPTL4_ following stimulation with DSS, SA, IL-1β, TNF-α, HC or NaBu. (**h**) Decay curves of ANGPTL4 and TTP in iCEC_ANGPTL4_, iCEC_TTP_ or iCEC_Ctrl_, following actinomycin D treatment after NaBu stimulation. (**i**) Transwell migration assay set-up. siRNA knockdown was performed on iCECs seeded in wells while THP1-derived macrophages were cultured on inserts. The macrophages were later introduced to iCECs and further stimulated with either pro- or anti-inflammatory stimuli. The Mann–Whitney U test was used. *p < 0.05, **p < 0.01, ***p < 0.001.


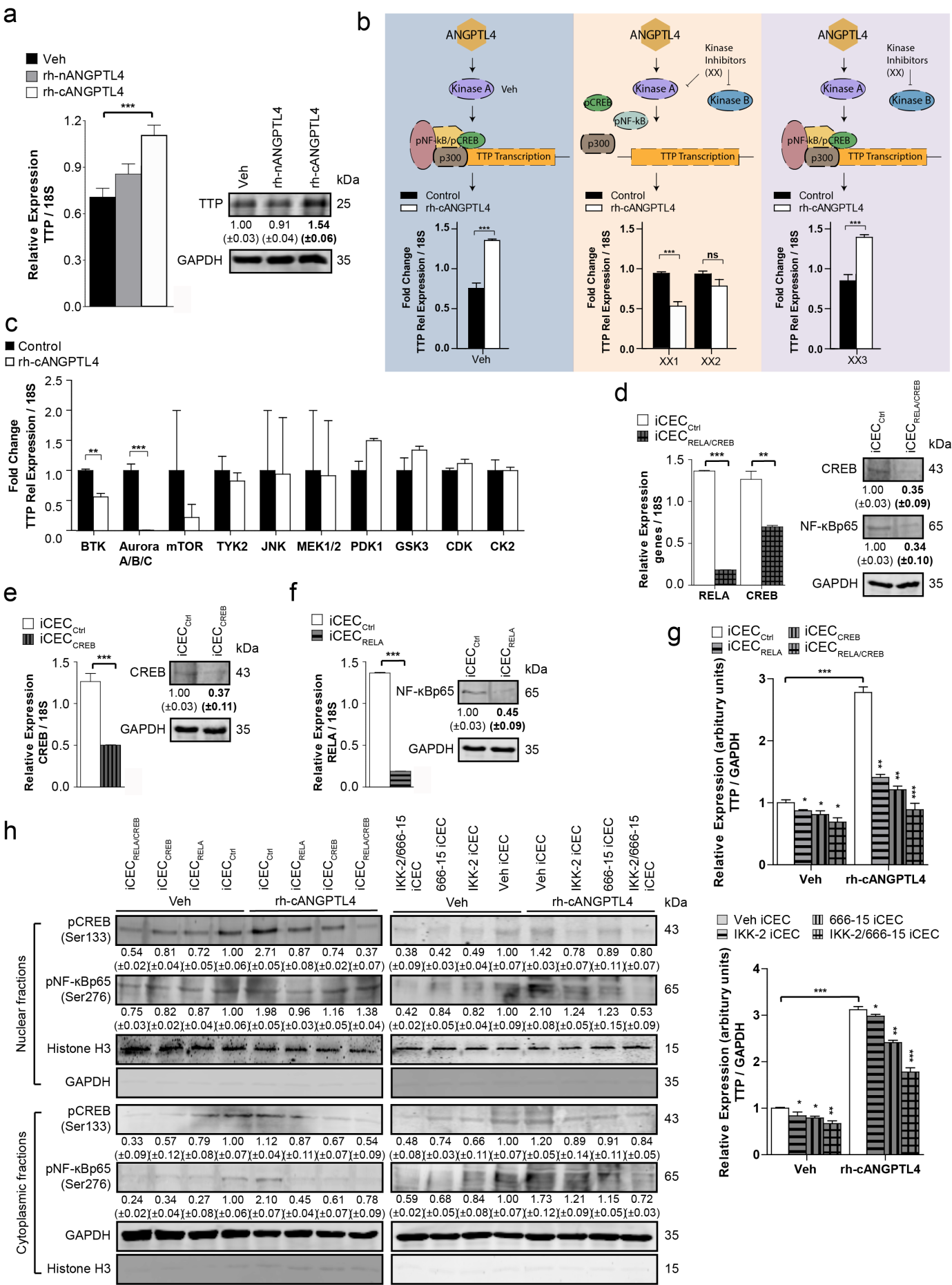


**Supplementary Fig. S3. Expression of TTP transcript in iCECs treated with various kinase inhibitors.**

**(a)** Relative mRNA (*left panel*) and protein (*right panel*) levels accounting for TTP expression in iCECs treated with rh-cANGPTL4 and rh-nANGPTL4. **(b)** A schematic illustration of the rationale behind the kinase inhibitor array, which led up to mapping the IPA pathway in Fig. 4a. When treated with rh-cANGPTL4, TTP expression was increased in iCECs (*left panel*). Kinase inhibitors (XX_n_) that negated this up-regulation of TTP in the presence of rh-cANGPTL4 meant that these kinases were directly responsible for regulating TTP expression (*middle panel*). On the other hand, the addition of kinase inhibitors that brought about an increase in TTP expression in the presence of rh-cANGPTL4 (*right panel*) suggested that ANGPTL4 works through other unique pathways to up-regulate the expression of TTP, independent of the target kinase. (**c**) Relative mRNA level of TTP after treatment with various kinase inhibitors from a SYNLibrary 95 array. Relative mRNA (*left panel*) and protein (*right panel*) levels accounting for the levels of (**d**) RELA and CREB, **(e)** CREB and (**f**) RELA following siRNA knockdown as determined by real-time PCR and immunoblot analysis, respectively. (**g**) Relative expression of TTP in iCECs after the depletion of CREB and/or NF-κB using siRNA knockdown (iCEC_Ctrl_, iCEC_RELA,_ iCEC_CREB_ and iCEC_RELA/CREB_) and CREB and/or NF-κB inhibitors (Veh iCEC, IKK-2 iCEC_,_ 666-15 iCEC and IKK-2 /666-15 iCEC). (**h**) Immunoblot analysis of pCREB, CREB, and pNF-κB for nuclear (*upper panel*) and cytoplasmic fractions (*lower panel*) isolated from iCECs (iCEC_Ctrl_, iCEC_RELA,_ iCEC_CREB_, iCEC_RELA/CREB_, Veh iCEC, IKK-2 iCEC_,_ 666-15 iCEC and IKK-2 /666-15 iCEC) stimulated with or without rh-cANGPTL4. Mann–Whitney U test was used. Three independent experiments (n = 3) were performed (means ± s.e.m.); *p < 0.05, **p < 0.01, ***p < 0.001.

**
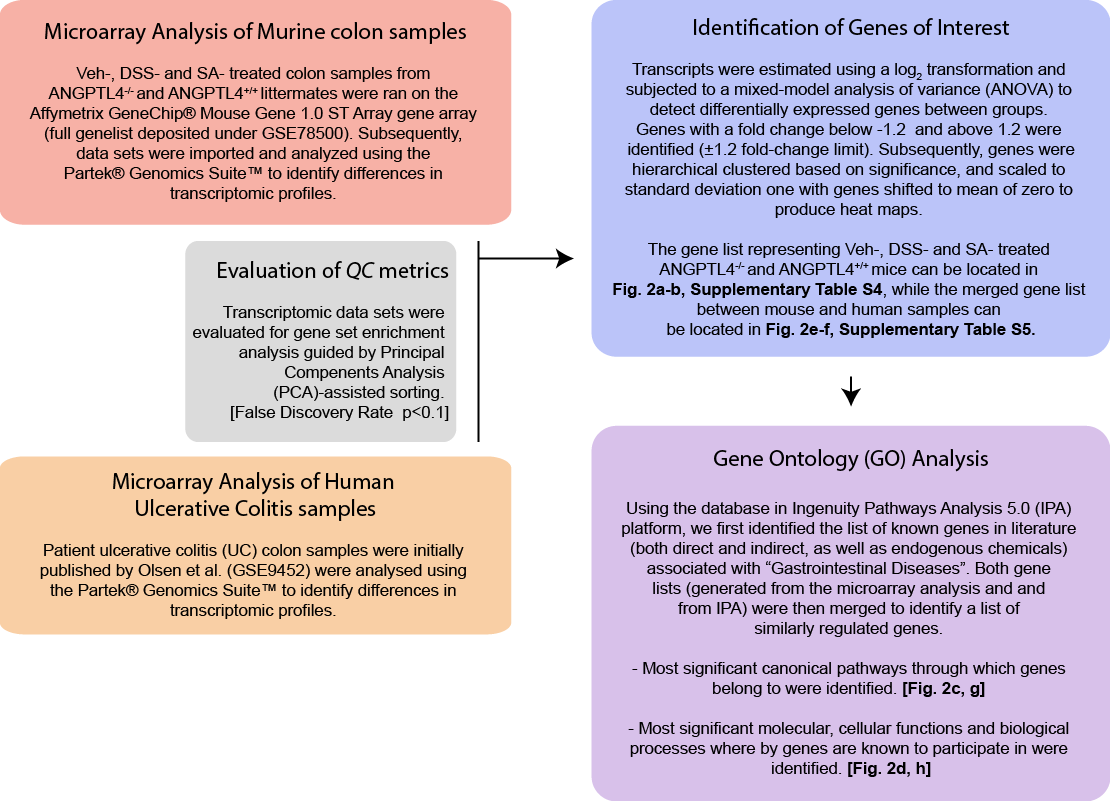
**

**Supplementary Fig. S4. Schematics of the microarray analysis.**

Schematics illustrating the flow of the microarray analysis. Both murine and human samples were subjected to ANOVA in Partek Genomics Suite software to evaluate and identify changes in gene transcriptomic profiles. Subsequently, datasets were analyzed using Ingenuity Pathway Analysis to identify top hits in canonical pathways and biological processes.
